# Structures and implications of the nuclease domain of human parvovirus B19 NS1 protein

**DOI:** 10.1016/j.csbj.2022.08.047

**Published:** 2022-08-27

**Authors:** Yixi Zhang, Zhiwei Shao, Yanqing Gao, Boming Fan, Jie Yang, Xi Chen, Xin Zhao, Qiyuan Shao, Weizhen Zhang, Chulei Cao, Hehua Liu, Jianhua Gan

**Affiliations:** Shanghai Public Health Clinical Center, State Key Laboratory of Genetic Engineering, Collaborative Innovation Center of Genetics and Development, Department of Biochemistry and Biophysics, School of Life Sciences, Fudan University, Shanghai 200438, China

**Keywords:** Human parvovirus B19, B19V, NS1 nuclease domain, Crystal structure, DNA binding, DNA cleavage, B19V, Human parvovirus B19, NS1_Nuc, Nuclease domain of NS1, ssDNA, single -stranded DNA, dsDNA, double -stranded DNA, NSBE, NS1-binding element, TRS, terminal resolution site, ITR, identical terminal repeat, AAV5, Adeno-associated virus type 5, PCV2, Porcine circovirus 2, WDV, Wheat dwarf virus

## Abstract

Infection of human parvovirus B19 (B19V) can cause a variety of diseases, such as hydrops fetalis, erythema infectiosum in children and acute arthropathy in women. Although B19V infection mainly occurs during childhood, about 50 % of adults are still susceptible to B19V infection. As the major replication protein of B19V, deletion of NS1 completely abolishes the infectivity of the virus. The nuclease domain of NS1 (NS1_Nuc) is responsible for DNA Ori binding and nicking that is critical for B19V viral DNA replication. NS1 has various variants, the structure and function for the majority of the variants are poorly studied. Here, we report two high-resolution crystal structures of NS1_Nuc, revealed the detailed conformations of many key residues. Structural comparison indicates that these residues are important for ssDNA or dsDNA binding by NS1. NS1 belongs to the HUH-endonuclease superfamily and it shares conserved ssDNA cleavage mechanism with other HUH-endonuclease members. However, our structural analyses, mutagenesis and *in vitro* assay results all suggested that NS1_Nuc utilizes one unique model in ssDNA binding.

## Highlights

• Two NS1 nuclease domain structures were determined at atomic resolution.
• The detailed conformations and/or conformational changes were revealed.
• Residues important for NSBE element binding were identified.
• NS1 uses one unique model in target ssDNA recognition.

## Graphical abstract

**Figure:**
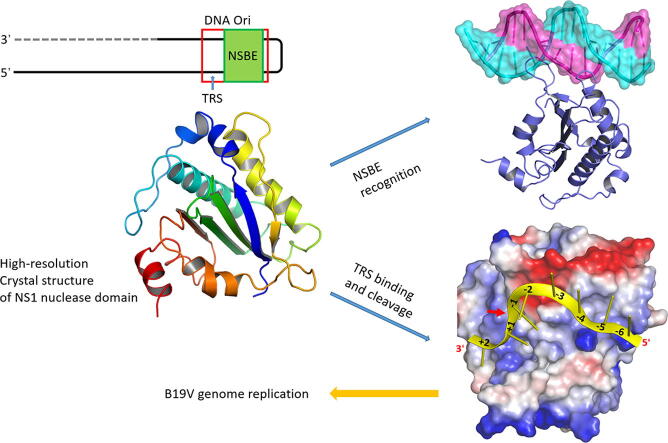

## 1 Introduction

Human parvovirus B19 (B19V) is a small non-enveloped DNA virus, and it belongs to the erythrovirus genus within the *Parvoviridae* family [1]. Like hepatitis B virus, hepatitis C virus and type 1 human immunodeficiency virus, B19V is a blood-borne virus [2], which can be transmitted by blood and blood products [3], [4], [5], [6]. B19V can also be transmitted via respiratory droplets [7], hand-to-mouth contact, organ transplantation [8], [9] and vertically from mother to the fetus [10]. Although B19V infection mainly occurs during childhood, about 50 % of adults are still susceptible to B19V respiratory droplet infection [2], [11]. Infection of B19V has been linked with a variety of diseases [12], such as hydrops fetalis (a serious condition of the fetus) [13], erythema infectiosum (also known as the fifth disease) in children [14], acute arthropathy in women and transient aplastic crisis in patients with chronic hemolytic anemia [15]. In addition, B19V is also one of the most common causes of myocarditis [16], a life-threating condition in pediatric patients.

**Fig. 1:**
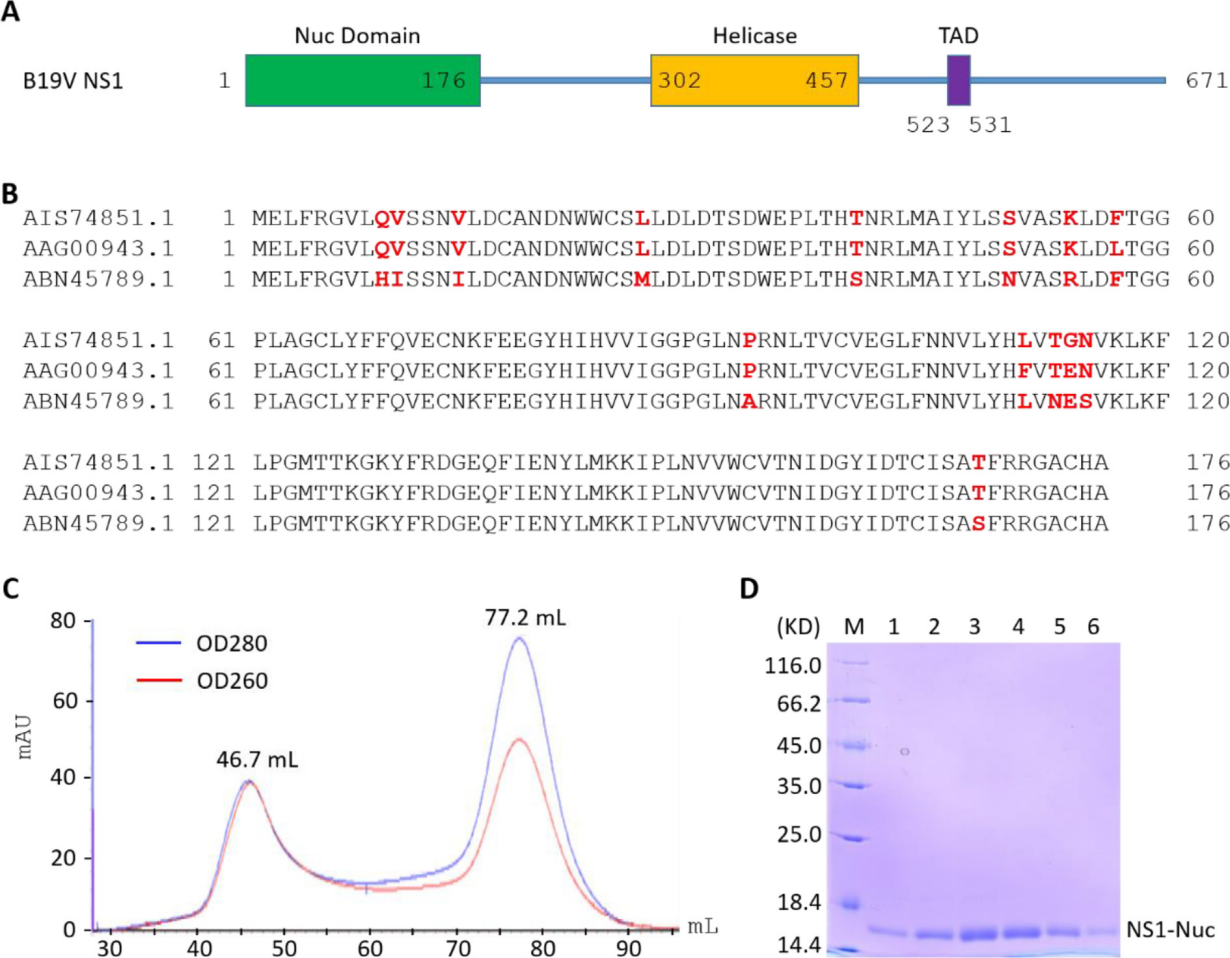
Purification of the NS1_Nuc protein. A) The domain architecture of NS1 protein. B) Sequence alignment of three NS1_Nuc domain derived from different NS1 variants. C) Size-exclusion chromatographic profile of the NS1_Nuc protein. D) SDS-page gel analysis of the NS1_Nuc protein.

B19V is a single-stranded DNA (ssDNA) virus with a genome of 5596 nucleotides (nts). The central region of B19V genome encodes for six proteins: capsid proteins VP1 and VP2, non-structural protein NS1, two smaller non-structural proteins of 7.5 kDa and 11 kDa, and one additional protein with unknown function [17], [18]. The 11 kDa protein is involved in viral replication and interaction with host protein Grb2 [19], [20], while the function of the 7.5 kDa protein is unclear. NS1 is the major replication protein; it predominantly localizes in the nucleus of infected cells. NS1 is of 671 amino acids (aa) in size with a molecular weight of 78 kDa (Fig. 1A). NS1 contains one nuclease domain (Nuc, aa 1–176), one helicase domain (aa 302–457) and one transactivation domain (TAD, aa 523–531). The TAD domain is critical for the promoter transactivation activity of NS1 and arrest of the infected primary erythroid progenitor cells at G2 phase [21]. With the assistance of transcription factors Sp1/Sp3, NS1 can bind and regulate the expression of its own gene and several genes of the host organism [21], [22], [23].

NS1 plays important role in the replication of B19V genome, which follows a “rolling hairpin” mechanism. The hairpin structures are formed by the identical terminal repeats (ITRs) within the left and right ends of the genome. During replication, one cellular DNA polymerase extends the 3’-end of the terminal hairpin, replicating the majority of the genome [24]. Replication of the remaining genome requires a new 3’-end, which is generated by NS1 cleavage at the terminal resolution site (TRS) within the ITR sequences [20], [25]. Adjacent to TRS, there are four NS1-binding elements, namely NSBE1 to NSBE4, which are necessary for optimal replication of the virus [26]. Previous studies has identified one minimal replication origin (Ori), which is 67-nt in size and contains both the TRS and NSBE elements [26].

The nuclease domain of NS1 (NS1_Nuc) is responsible for B19V Ori DNA binding and nicking [27]. NS1 has various variants with significant sequence divergence, especially at the nuclease domain region. The structural studies of NS1 are very limited. To date, no DNA-complexed structure is available for any NS1 variant (either full-length or the nuclease domain), which hindered our understanding on target DNA recognition and cleavage by NS1. Very recently, the apo-form structures were reported for the nuclease domains of two NS1 variants [28]; however, due to resolution limitation, many key residues were not observed. Here, we report the structures and biochemical studies of NS1_Nuc from one different variant. The structures were refined at atomic resolution, revealed the detailed conformations of several key residues. In addition to structural analyses, our biochemical assay results also support one novel NS1_Nuc/ssDNA binding model. Our study advances our understanding on the structure and function of NS1.

## 2 Materials and methods

### 2.1 Plasmid construction

The codon-optimized cDNA (Supplementary Table S1) of NS1_Nuc was ordered from Shanghai Generay Biotech Co., ltd, China. The target region was amplified by polymerase chain reactions (PCR), cleaved by *Bam*HI and *Xho*I, and cloned into pET28a-Sumo vectors. The recombinant plasmids of NS1_Nuc mutants were constructed by overlap PCR using the wild-type (WT) NS1_Nuc plasmid as template and primers listed in Supplementary Table S2. Sequences of WT and mutant plasmids were confirmed by DNA sequencing. The recombinant plasmids were transformed into *E. coli* Rosetta (DE3) competent cells for protein expression.

### 2.2 Protein expression and purification

Both WT and mutant NS1_Nuc proteins were expressed and purified using similar procedures. The cells were cultured at 37℃ in LB medium. When the OD_600_ reached 0.6–0.8, isopropyl-D-1-thiogalacto-pyranoside (final concentration of 0.2 mM) was added to induce the expression of the proteins. The cultures were incubated at 16℃ for an additional 18–20 h. For expression of Seleno-methionine (Se-Met) substituted NS1_Nuc protein, cells were cultured in M9 medium supplemented with 30 mg/L l-selenomethionine.

The cells were collected via centrifugation and resuspended in Buffer A (20 mM Tris-HCl pH 8.0, 500 mM NaCl, 25 mM imidazole). The cells were lysed by high-pressure homogenization and centrifuged at 16000 rpm for 1 h at 4℃. The supernatant was applied to 5-mL HisTrap^TM^ HP column (Cytiva), and the target protein was eluted via AKTA purifier (GE Healthcare) using Buffer B (20 mM Tris-HCl pH 8.0, 500 mM NaCl, 500 mM imidazole). The target protein was pooled, treated with ULP1 protease, and dialyzed against Buffer C (20 mM Tris-HCl pH 8.0, 500 mM NaCl) overnight at 4℃. The sample was reloaded onto the HisTrap^TM^ HP column. The target protein was collected and applied to a Hiload 16/600 Superdex G75 column gel filtration column (Cytiva) equilibrated with Buffer D (20 mM Tris-HCl pH 8.0, 150 mM NaCl, 2 mM DTT). The purified protein was concentrated to 15 mg/ml and stored at −80 °C.

To analyze the molecular weight of NS1_Nuc, 500 μL proteins (300 μg) were applied to Superdex 200 Increase 10/300 GL column (GE Healthcare) equilibrated with Buffer D. The flow rate was set at 0.4 mL/min. Besides NS1_Nuc proteins, we also performed size-exclusion chromatography analysis for 100 μL standard marker proteins using the same column equilibrated with 20 mM Tris-HCl pH 7.0 and 150 mM NaCl buffer.

### 2.3 *In vitro* DNA binding assays

WT or mutant B19V NS1-Nuc protein (1–128 μM) was mixed with 0.1 μM DNA in binding buffer (100 mM Tris pH 8.0, 150 mM NaCl, 1 mM EDTA, 1 mM DTT and 10 % glycerol). The 5′-end of the 67-ori-top DNA strand is FAM-labeled. The samples were incubated at 4 °C for 2 h and then analyzed on 10 % native PAGE gels with 0.5 × TBE buffer. The gel was imaged using Typhoon FLA 9000. Intensities of the DNA bands were quantified by ImageQuantTJ. The percentage of binding, for each protein concentration, was calculated. Data were then fitted to the equation *Y* = B_max_*X^h/(K_d_^h + *X*^h) using nonlinear regression (curve fit) in GraphPad Prism. The dissociation constants (K_d_) were determined from the regression curve.

### 2.4 *In vitro* DNA cleavage assays

DNA cleavage assays were performed using 5′-FAM-labeled ssDNA (0.1 μM) and WT or mutant B19V NS1-Nuc protein (5 μM). Reaction mixtures were incubated at 37 °C in buffer composed of 50 mM HEPES pH 7.5, 150 mM NaCl, 10 mM MnCl_2_ and 10 % glycerol. The reactions were quenched by adding the termination buffer (90 % formamide, 20 mM EDTA, 0.05 % bromophenol blue and 0.05 % xylene blue) at various time points. Samples were loaded onto pre-warmed 16 % denaturing PAGE gels. The gel was visualized using Typhoon FLA 9000. Intensities of the substrate and product bands were quantified by ImageQuantTJ. The percentage of cleavage, for each protein concentration, was calculated.

### 2.5 Crystallization and data collection

The initial crystallization conditions were identified at 16 °C using the Gryphon crystallization robot system from Art Robbins Instrument company and crystallization kits from Hampton Research company. The sitting-drop vapor diffusion method with the 3-drop intelliplate plates were utilized during initial screening. The Form I crystals were optimized using the hanging-drop vapor diffusion method, the crystallization condition is composed of 1.0 M Ammonium phosphate (dibasic) and 0.1 M Sodium acetate/Acetic acid pH 4.2 buffer. The Form II crystals grew in 1.26 M Ammonium sulfate (dibasic) and 0.1 M HEPES/Sodium hydroxide pH 7.5 buffer, using sitting-drop vapor diffusion method. Each drop contains 0.4 μL protein sample and 0.2 μL crystallization buffer.

All crystals were cryo-protected by their mother liquid supplemented with 25 % glycerol and flash-frozen using liquid nitrogen. The diffraction data were collected on beamline BL18U1 at the Shanghai Synchrotron Radiation Facility (SSRF). Data processing was carried out using the HKL3000 program [29]. The data collection and processing statistics were summarized in Table 1.

**Table 1:** Data collection and refinement statistics.

Structure	Form I	Form II
PDB ID	7Y56	7Y57

**Data collection ^a^**		
Space group	*P*4_1_2_1_2	*P*2_1_
Cell parameter:		
α, *b*, *c* (Å)	69.4, 69.4, 108.9	74.0, 74.0, 186.5
α, β, γ (°)	90.0, 90.0, 90.0	90.0, 90.0, 90.0
Wavelength (Å)	0.9793	0.9793
Resolution (Å)	50.0–1.75	50.0–2.2
High-resolution shell (Å)	1.78–1.75	2.24–2.2
Completeness (%)	100.0 (100.0)	98.1 (84.0)
Redundancy	13.6 (12.4)	3.2 (2.3)
R_merge_ (%)	9.5 (55.8)	10.5 (38.2)
I/σ(I)	23.1 (3.4)	8.1 (1.6)

**Refinement**		
Resolution (Å)	32.2–1.75	43.4–2.2
No. of reflections	27,340	18,010
R_work_ (%) **/** R_free_ (%)	18.2/20.8	22.3/25.8
No. of atoms		
Protein	1402	2732
Water	184	41
R.m.s. deviations		
Bond length (Å)	0.007	0.003
Bond angle (°)	0.840	0.702
Ramachandran plot (%)		
Most favorable	96.5	97.0
Additional allowed	3.5	3.0
Outlier	0.00	0.0

a: Values in parentheses are for the high-resolution shell.

### 2.6 Structure determination and refinement

The Form I NS1_Nuc structure was solved by the single-wavelength anomalous diffraction method [30] with the Autosol program embedded in the Phenix suite [31]. The initial model was built using the Autobuilt program and then refined against the diffraction data using the Refmac5 program of the CCP4 suite [32]. The 2F_o_ –F_c_ and F_o_ –F_c_ electron density maps were regularly calculated and used as guide for the building of the missing amino acids using COOT [33]. The Form II structure was solved by molecular replacement method using the Form I structure as the search model with the phaser program of the CCP4 suite [34]. The final refinement of both structures was performed using the phenix.refine program of Phenix suit. The structural refinement statistics were also summarized in Table 1.

### 2.7 Data deposition

The coordinate and structure factors have been deposited in the Protein Data Bank under accession codes 7Y56 and 7Y57 for the Form I and Form II NS1_Nuc structures, respectively.

## 3 Results and discussion

### 3.1 Design of a novel NS1_Nuc construct

Among the reported NS1_Nuc structures, one (PDB_ID: 6SUM) is derived from the NS1 variant with GenBank accession number of AAG00943.1, whereas the other structures (PDB_ID: 7SZX and 7SZY) are all derived from the variant with GenBank accession number of ABN45789.1. Out of the 176 amino acids of the NS1_Nuc domain, 13 are different between the two variants (Fig. 1B). In this study, we mainly focus on the nuclease domain of NS1 variant with GenBank accession number of AIS74851.1. Compared to the reported NS1_Nuc proteins, our NS1_Nuc has 3 and 13 amino acid substituted, respectively (Fig. 1B).

In addition to the NS1_Nuc domain, the 6USM structure contains one maltose binding protein tag at the *N*-terminus. The protein utilized for the crystallization of the 7SZX structure has 20 and 33 extra residues at the N- and C-termini, respectively. The protein contains 6 extra residues at the *N*-terminus for the 7SZY structure. Different from previous studies, we designed one His-Sumo-NS1_Nuc construct. The His-Sumo Tag was included to enhance expression and solubility of the protein. During purification, the His-Sumo-tag was removed by protease ULP1, only leaving one Gly and one Ser residue at the *N*-terminus of the target protein.

The target NS1_Nuc protein has an elution volume of 77.2 mL on Hiload 16/600 Superdex G75 column (Fig. 1C). The OD280/OD260 value is close to 1.6, indicating that the protein is free of nucleic acid contamination. The SDS-Page gel analysis confirmed that the protein was purified to homogeneity (Fig. 1D). The theoretic molecular weight of NS1_Nuc is 19.8kD, but it moves faster than the 18.8kD marker on the SDS-Page gel. Puzzled by these observations, we performed size-exclusion analysis for NS1_Nuc and the standard marker proteins using the Superdex 200 Increase 10/300 GL column. The apparent molecular weight of NS1_Nuc is also smaller than the theoretic value (Supplementary Fig. S1). The reason for the abnormal movement of NS1_Nuc is unclear at present.

### 3.2 NS1_Nuc possesses DNA binding and cleavage activity

**Fig. 2:**
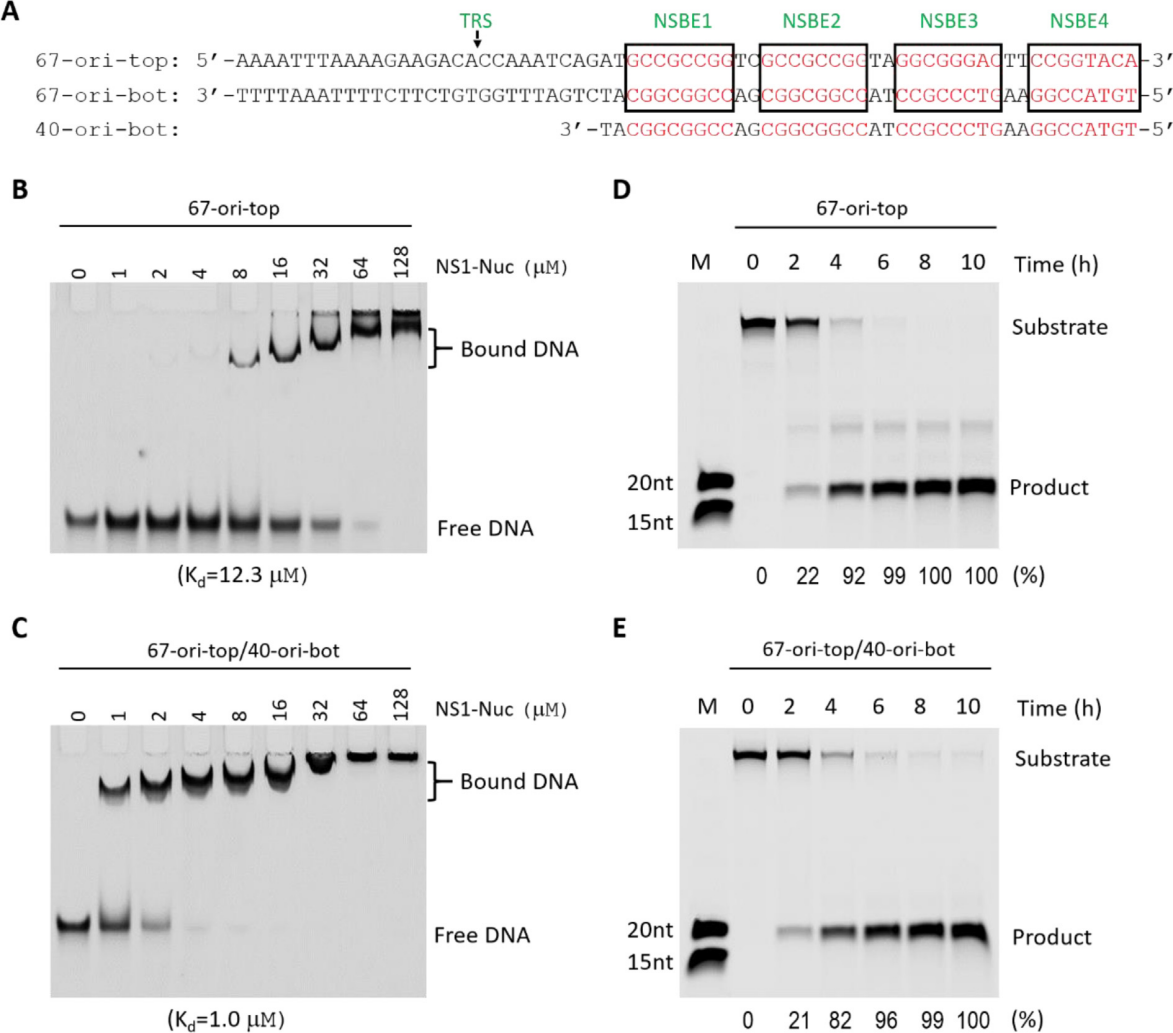
DNA binding and cleavage by the NS1_Nuc protein. A) Sequence of the identified DNA Ori of B19V. The TRS site is indicated by black arrow. The four NSBE elements are highlighted with black boxes. B-C) *In vitro* DNA binding by the NS1_Nuc protein. D-E) *In vitro* DNA cleavage assays catalyzed by the NS1_Nuc protein. The dissociation constant (K_d_) values and substrate cleavage percentage derived from three independent experiments are listed at the bottom of the figures.

The 67-bp Ori DNA of B19V is composed of two strands, 67-ori-top and 67-ori-bot (Fig. 2A). NS1_Nuc derived from the variant with GenBank accession number of ABN45789.1 can bind the duplex DNA, but it only cleaves 67-ori-top in single-stranded form. The *in vitro* DNA binding and cleavage activities have not been confirmed for other NS1 variants, including the variant with GenBank accession number of AAG00943.1 and the one studied in this work, which are more similar in sequence (Fig. 1B). Using the purified NS1_Nuc protein, we first performed *in vitro* DNA-binding assay. As depicted in Fig. 2B, the protein can bind the single-stranded 67-ori-top DNA with a dissociation constant (K_d_) value of 12.3 μM. Adding 40-ori-bot strand, which contains the sequence complementary to NSBE1-NSBE4, can significantly enhance the DNA binding affinity of the protein (Fig. 2C); the K_d_ value is approximately 1.0 μM.

We then performed *in vitro* DNA cleavage assays. As depicted in Fig. 2D-E, NS1_Nuc protein can cleave the single-stranded 67-ori-top DNA and 67-ori-top/40-ori-bot mixture at similar rate. Comparison with the DNA markers (15nt and 20nt) indicated that the 67-ori-top DNA was cleaved at the correct site; the target products are 18nt in size. The *in vitro* DNA cleavage activity of NS1_Nuc is weak. At a reaction time of 2 h, only about 20 % DNA substrates were cleaved; and, about 10 % substrates still remained intact at a reaction time of 4 h. Similar phenomenon was also observed in previous studies. There are two possible explanations for the low cleavage activity of the NS1_Nuc proteins. Firstly, instead of Mn^2+^, NS1 may utilize other cations in substrate DNA coordination and cleavage *in vivo*. Secondly, the other domains of NS1, especially the helicase domain, may help substrate binding and correct orientation, increasing the DNA cleavage activity of NS1.

### 3.3 Crystal structures of NS1_Nuc

**Fig. 3:**
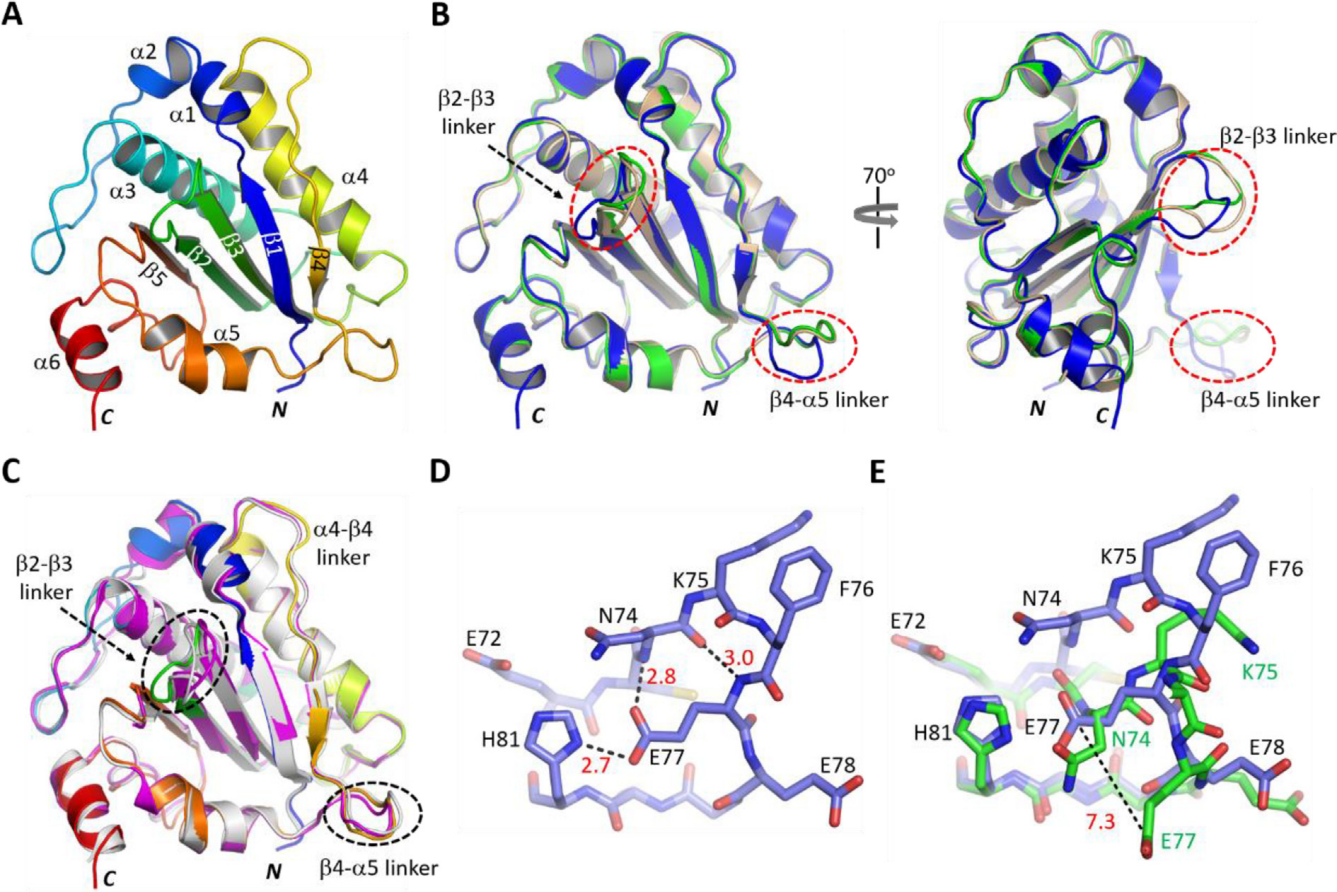
Crystal structures of the NS1_Nuc protein. A) Cartoon presentation of the Form I structure. B) Superposition of the Form I and Form II NS1_Nuc structures, which are colored in blue and wheat, respectively. C) Superposition of the Form I NS1_Nuc structure with the 6USM and 7SZY structures. The two latter structures are colored in white and magenta, respectively. D) The detailed conformations of the β2-β3linker observed in the Form I NS1_Nuc structure. E) Structural superposition showing the conformational changes of the β2-β3linker. The C-atoms are colored in light blue and green in the Form I and Form II structures, respectively. The distances (Å) are shown in numbers. (For interpretation of the references to colour in this figure legend, the reader is referred to the web version of this article.)

Upon confirmation of its DNA binding and cleavage activities (Fig. 2B-E), we then performed crystallization trials for the NS1_Nuc protein. Totally, two NS1_Nuc structures (Form I and Form II) were solved. Compared to the 6USM (3.5 Å), 7SZX (3.5 Å) and 7SZY (2.4 Å) structures, the resolution of the Form I structure is much higher (1.75 Å, Table 1). The Form I structure belongs to *P*4_1_2_1_2 space group; and, it was refined to final R_work_ and R_free_ of 18.0 % and 22.2 %, respectively. There is one NS1_Nuc molecule within the asymmetric unit. Except the extra Gly residue at the *N*-terminus and His175 and Ala176 at the C-terminus, all other residues are well ordered in the structure. As depicted in Fig. 3A, NS1_Nuc is of α/β fold in nature. The five strands (β1-β5) form one flat antiparallel β-sheet in the center, flanked by four helices (α1-α6) on one side and another two helices (α5-α6) on the opposite side.

The Form II structure belongs to *P*2_1_ space group. It was refined to 2.2-Å resolution with final R_work_ and R_free_ of 22.3 % and 25.8 %, respectively. Different from the Form I structure, there are two NS1_Nuc molecules within the asymmetric unit of the Form II structure. Superposition showed that the overall folding of NS1_Nuc is very similar in the two structures (Fig. 3B); the root mean square deviation (RMSD) value is 0.8 Å, based on 169 pairs of Cα atoms. The conformations of the α-helices and -strands are well conserved, but NS1_Nuc does show some conformational changes, especially in the βlinker and β4-β5 linker regions.

The NS1_Nuc protein also shares similar fold with the 6USM and 7SZY structures [28]; the RMSD values among them are around 0.9 Å (based on 162 and 169 pairs of Cα atoms, respectively). In addition to the β2-β3linker and β4-β5 linker, structural superposition (Fig. 3C) also revealed conformational changes for other regions of NS1_Nuc, such as the α4-β4linker. The most distinctive conformation was observed for the β2-β3linker in the Form I structure (Fig. 3D). The main chain of Glu77 forms one hydrogen bond (H-bond) interaction with Asn74. The side chain of Glu77 forms two H-bond interactions: one with the main chain of Asn74 and the other with the side chain of His81 (one of the catalytic residues). Compared to the Form II structure, Glu77 is shifted about 7.3 Å in the Form I structure (Fig. 3E). However, mutagenesis and *in vitro* assay results suggested that Glu77 is not involved in direct DNA binding and cleavage by NS1_Nuc (Supplementary Fig. 2).

### 3.4 Identification of NSBE-interacting residues

**Fig. 4:**
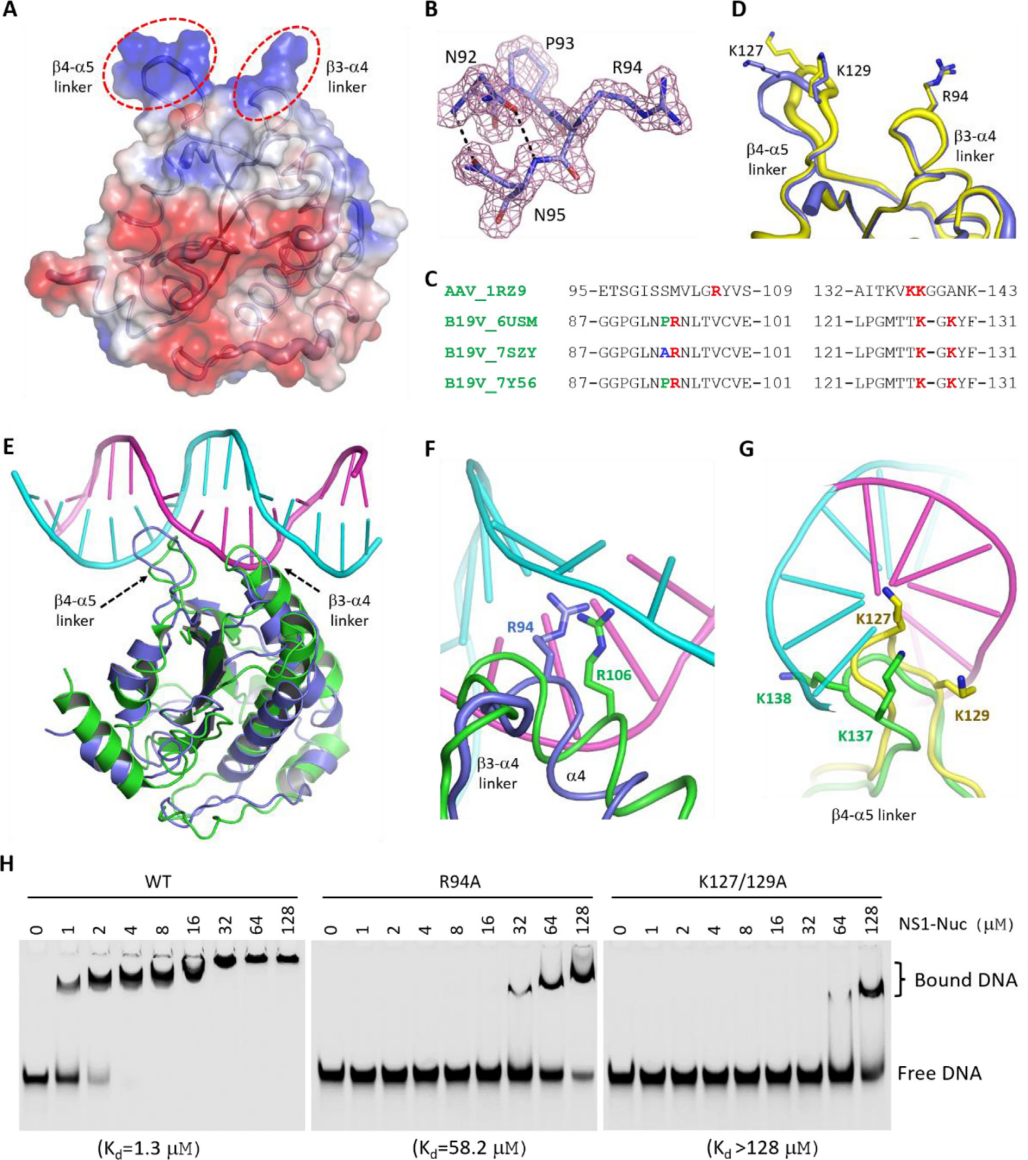
Identification of NS1 residues important for NSBE element binding. A) Cartoon and surface presentation of the Form I NS1_Nuc structure. The electrostatic surface is calculated by Pymol with the default settings. B) The detailed conformations and 2F_o_-F_c_ electron density maps (contour level, 1.5σ) of the β3-α4 linker. C) Sequence alignment of NS1 variants and AAV5 Rep protein. D) Superposition of the β3-α4 and β4-α5 linkers in the Form I and Form II NS1_Nuc structures. E) Superposition of Form I NS1_Nuc structure with the AAV5 Rep/dsDNA complex (PDB_ID: 1RZ9). F) Conformational comparison of NS1 Arg94 with Arg106 of AAV5 Rep. G) Superposition of NS1_Nuc β4-α5 linker and the corresponding linker of AAV5 Rep. C-atoms are colored in light blue and yellow in the Form I and Form II NS1_Nuc structures, respectively, in all panels. H) Comparison of dsDNA binding by WT and mutant proteins of NS1_Nuc. The K_d_ values derived from three independent experiments are listed at the bottom of the figures. (For interpretation of the references to colour in this figure legend, the reader is referred to the web version of this article.)

Out of the 176 residues of our NS1_Nuc protein, 14 are Lys or Arg, forming several positive electrostatic patches on the surface of the protein (Fig. 4A). Two of the patches are very close in space. One is composed of the β3-α4linker, the other is formed by the β4-αlinker. As depicted in Fig. 4B, Asn92 and Asn95 of the β3-α4linker form two H-bonds, via their main chain N atoms and side chain OD1 atoms. Different from the 7SZY structure, which has one Ala residue at position 93, our NS1_Nuc structure has one Pro residue at the corresponding position (Fig. 4C). Pro93 is very rigid, which may enhance the conformational stability of the3α4linker. Arg94 is highly conserved in NS1 variants (Fig. 4C). The side chain of Arg94 was not observed in any reported NS1_Nuc structures, might be due to their resolution limitation. However, as supported by the clear 2F_o_-F_c_ electron density maps (Fig. 4B), Arg94 is well defined and adopts similar conformations in our NS1_Nuc structures (Fig. 4D). The β4-α5linker contains two highly conserved Lys residues, Lys127 and Lys129 (Fig. 4C). Different from Arg94, both Lys127 and Lys129 can undergo subtle conformational changes (Fig. 4D).

The Ori DNA of B19V contains four NSBE elements (Fig. 2A). Our *in vitro* binding assay showed that formation of NSBE duplexes can enhance target DNA binding by NS1_Nuc (Fig. 2B-C). Currently, no NS1/dsDNA complex is available, but one dsDNA-complexed structure of Adeno-associated virus type 5 (AAV5) replication initiator protein nuclease domain (Rep_Nuc) has been reported [35]. The nuclease domains of B19V NS1 and AAV5 Rep are distantly related. As depicted in Fig. 4E, the overall structures of NS1_Nuc and AAV5 Rep_Nuc are similar. In addition, NSBEs of B19V Ori DNA (Fig. 2A) and the DNA bound by AAV5 Rep_Nuc are all rich in C and G nucleotides. Based on structural comparison, it was previously predicted that NS1_Nuc and AAV5 Rep_Nuc follow similar manner in dsDNA recognition. However, due to the disordering of the reported structures, the detailed NSBE-interacting residues of NS1-Nuc are not clear.

Superposition our structure with the AAV5 Rep_Nuc/dsDNA complex (PDB_ID: 1RZ9) can shade some light on NSBE binding by NS1 (Fig. 4E). Similar to AAV5 Rep_Nuc, NS1_Nuc mainly uses the positively charged residues from the β3-α4and β4-α5linker regions in NSBE interaction. The β3-α4linker of NS1_Nuc inserts into the minor grove of dsDNA. Although not identical in location (Fig. 4C), Arg94 of NS1_Nuc can mimic Arg106 of AAV5 Rep_Nuc, forming sequence-specific interactions with the NSBE elements (Fig. 4F). The β4-α5linker of NS1-Nuc and the corresponding linker of AAV5 Rep_Nuc all insert into the major groove of dsDNA (Fig. 4G). Lys137 and Lys138 of AAV5 Rep_Nuc interact with the nucleobase and backbone of the DNA, respectively. Structural superposition indicated that NS1_Nuc Lys127 can mimic AAV5 Rep_Nuc Lys137 in DNA nucleobase recognition. NS1_Nuc Lys129 is able to interact with DNA backbone; however, different from Lys138 of AAV5 Rep_Nuc, NS1_Nuc Lys129 recognizes the opposite DNA strand. To further support the NSBE-binding model of NS1_Nuc, we constructed two NS1_Nuc mutants (R94A and K127/129A) and performed *in vitro* DNA binding assays using 67-ori-top/40-ori-bot mixture (Fig. 4H). Compared to the WT NS1_Nuc protein, the DNA-binding affinities of the two mutants are significantly weaker, suggesting that Arg94, Lys127 and/or Lys129 play important roles in NSBE-binding by NS1_Nuc.

### 3.5 Comparison with other HUH-endonuclease structures

**Fig. 5:**
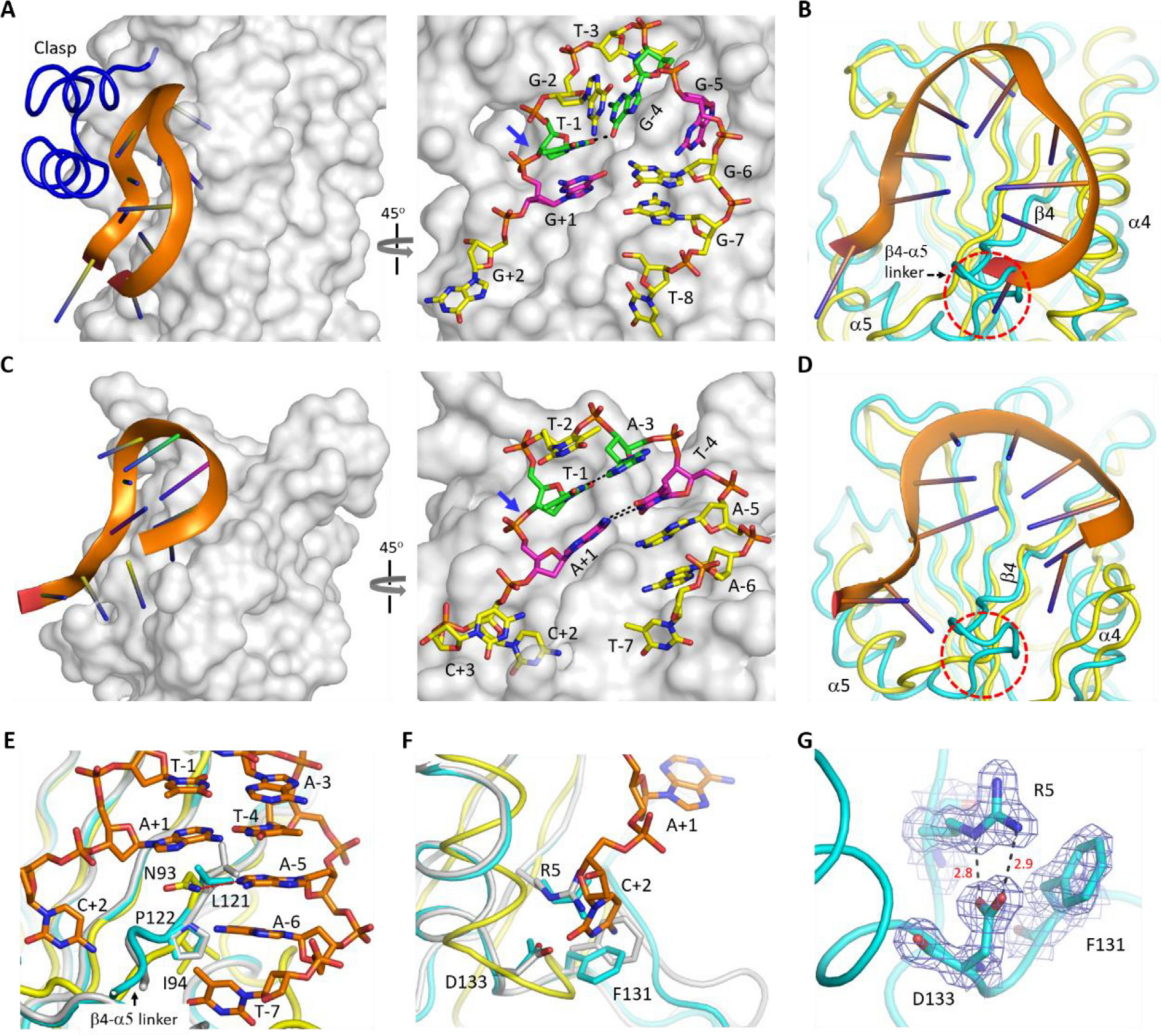
Structural comparison with other HUH-endonucleases. A) Crystal structure of TraI/ssDNA complex (PDB_ID: 2A0I). TraI protein is shown as surface with ‘clasp’ subdomain highlighted as cartoon in blue. ssDNA is shown as cartoon or sticks. B) Superposition of the core domains of NS1_Nuc and TraI relaxase. C) Crystal structure of WDV Rep/ssDNA complex (PDB_ID: 6WE1). D) Superposition of the core domains of NS1_Nuc and WDV Rep. E-F) Close view showing the clash between NS1 residues (Leu121, Pro122 and Arg5) and the nucleotides (A-5, A-6 and C + 2) of the DNA bound by WDV Rep. G) Conformation and stable interactions of Arg5 and Asp133 residues of NS1_Nuc. The 2F_o_-F_c_ electron density maps were contour at 1.5σ level. In panels B and D-G, C-atoms are colored in cyan and white in the Form I and Form II NS1_Nuc structures, respectively. (For interpretation of the references to colour in this figure legend, the reader is referred to the web version of this article.)

NS1 belongs to the HUH-endonuclease superfamily. Via binding and breakage of ssDNA, HUH-endonucleases participate in many fundamental biological processes such as rolling hairpin replication, rolling circle replication, DNA transposition and DNA integration into host genomes [36], [37], [38]. Reps and Relaxases are the two major classes of HUH-endonucleases. The ssDNA-complexed structures have been reported for several Reps and Relaxases, including Porcine circovirus 2 (PCV2) Rep [39], Wheat dwarf virus (WDV) Rep [39], relaxase TraI [40] and relaxase TrwC [41]. Though they share two conserved catalytic Histidine residues, the detailed structure and ssDNA binding mechanism of HUH-endonucleases are changeable. Besides the central catalytic domain, TraI (PDB_ID: 2A0I) and TrwC also contain one additional ‘clasp’ subdomain, which enhances the binding of the ‘*n*-shaped’ target ssDNA (Fig. 5A). The central catalytic domain has three nucleotide-binding pockets, recognizing the nucleobases of G-5, T-3 and G + 1, respectively. Structural superposition showed that the central β-sheets possess similar conformations in NS1_Nuc and TraI, whereas α4helix and the β4-α5linker adopt quite different conformations in the two structures (Fig. 5B). The corresponding regions are involved in target DNA −8 to −5 site nucleotide recognition in the TraI/ssDNA complex.

The size of WDV Rep (137 amino acids) is smaller than that of NS1_Nuc. In the reported WDV Rep/ssDNA complex structure (PDB_ID: 6WE1), the target DNA also adopts ‘*n*-shaped’ conformation (Fig. 5C). Like TraI, WDV Rep has three nucleotide-binding pockets, whereas they recognize the nucleotides at the −6, −4 and + 2 positions, respectively. Conformation of the target ssDNA is further stabilized by the Watson-Crick pairing between T-4 and A + 1 and the H-bond interaction between A-3 and T-1. Structural superposition showed that the conformations of α4helix and the β4-α5linker of NS1_Nuc are also different from the corresponding regions in the WDV Rep structure (Fig. 5D). The corresponding regions are involved in target DNA −7 to −4 site nucleotide recognition in the TraI/ssDNA complex.

Based on the WDV Rep/ssDNA complex structure, one similar ssDNA binding model was proposed for NS1_Nuc previously [28]. However, the sequences of the target DNAs are different for NS1_Nuc and WDV Rep. The core motif of NS1_Nuc DNA target is composed of G_-7_A_-6_A_-5_G_-4_A_-3_C_-2_A_-1_C_+1_C_+2_, whereas it is composed of T_-7_A_-6_A_-5_T_-4_A_-3_T_-2_T_-1_A_+1_C_+2_ for the DNA target of WDV Rep. In the WDV Rep/ssDNA complex structure, the side chain of Asn93 forms sequence-specific H-bond interaction with the nucleobase of A-5. The corresponding Leu121 residue of NS1_Nuc is hydrophobic. As observed in the Form I and Form II NS1_Nuc structures, Leu121 can adopt two different conformations, but neither are compatible with A-5 in the WDV Rep/ssDNA complex structure (Fig. 5E). Instead of flexible Ile, NS1_Nuc has one rigid Pro residue (Pro122) at the *N*-terminus of the β4-α5linker. The side chain of Pro122 is too close to allow A-6 binding in the identical orientation as observed in WDV Rep/ssDNA complex.

Both previous study [27] and our mutagenesis and *in vitro* assay results (Supplementary Fig. S3) confirmed that the core A_-1_C_+1_C_+2_ motif is important for target DNA cleavage by NS1_Nuc. In the WDV Rep/ssDNA complex structure, the nucleobase of C + 2 is inserted into a pocket and forms three H-bond interactions with the backbone O or N atoms of Ala96, Lys98 and Asp99. Due to sequence and conformational differences, the pocket was replaced by Arg5, Phe131 and Asp133 in the NS1_Nuc structure. The side change of Phe131 can undergo subtle conformational changes (Fig. 5F), but the conformations of Arg5 and Asp133 are well conserved, maintained by the stable H-bond interactions between their side chains (Fig. 5G). Structural superposition revealed serious clash between the nucleobase of C + 2 in the WDV Rep/ssDNA complex and Arg5 in the NS1_Nuc structure (Fig. 5F).

### 3.6 Proposed model for ssDNA binding and cleavage by NS1_Nuc

**Fig. 6:**
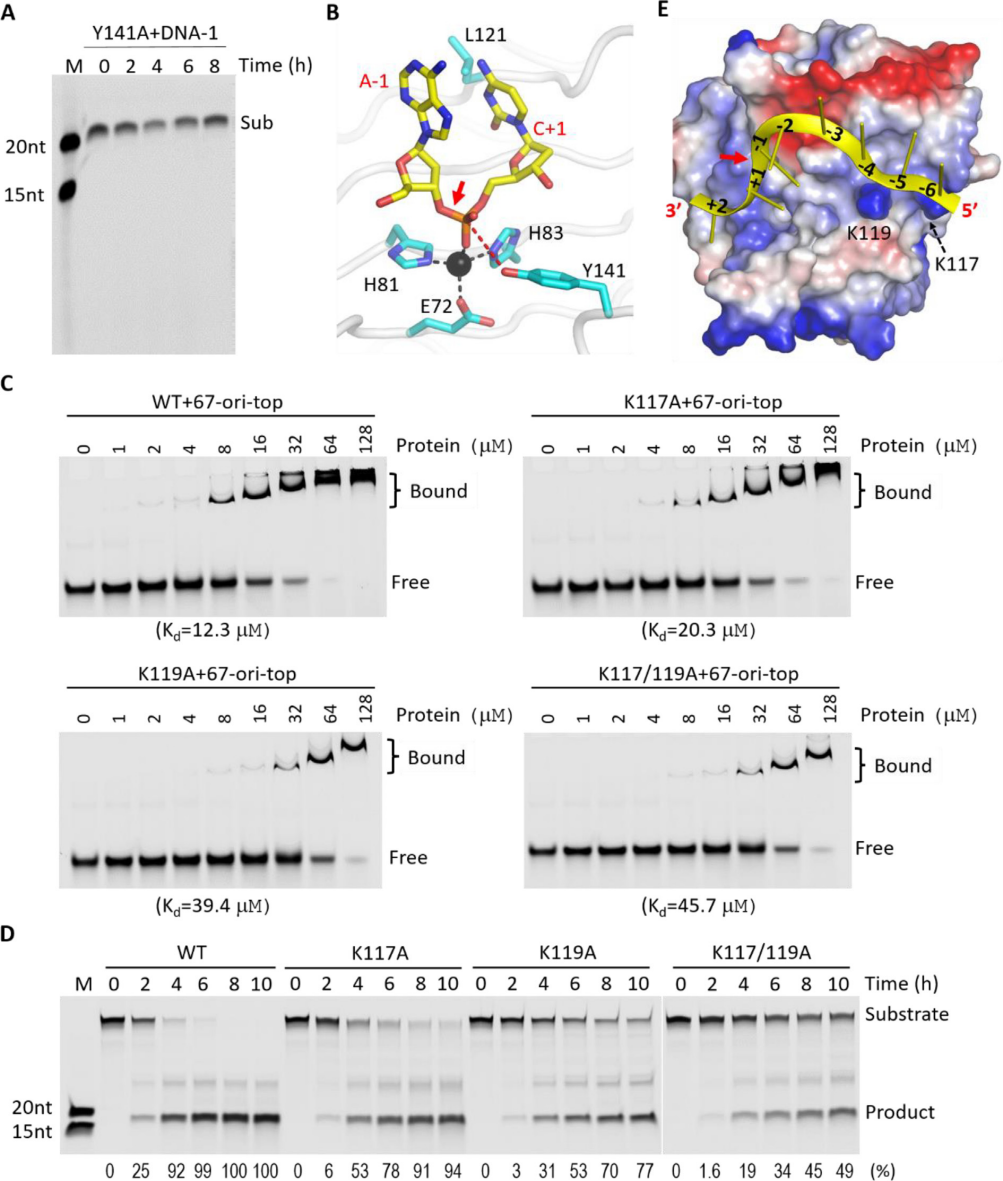
ssDNA binding and cleavage model for the NS1_Nuc protein. A) *In vitro* ssDNA cleavage assays catalyzed by Y141A mutant of NS1_Nuc. B) The proposed ssDNA cleavage model of NS1_Nuc. ssDNA and NS1 residues important for catalysis are shown as sticks. C-D) *In vitro* DNA binding and cleavage assays catalyzed by WT NS1_Nuc and mutants with single or double mutation of Lys117 and Lys119. The K_d_ values and substrate cleavage percentage derived from three independent experiments are listed at the bottom of the figures. E) The proposed ssDNA binding model of NS1_Nuc. The DNA and protein are shown as cartoon and surface presentation, respectively. The electrostatic surface is calculated by Pymol with the default settings.

The catalytic center of HUH-endonucleases is composed of four residues. Unlike the two characteristic Histidine residues, the third catalytic residue is changeable (either Glu or His). The fourth residue is a Tyrosine, which is highly conserved in HUH-endonucleases. To confirm the functional importance of the catalytic Tyr141 residue of NS1_Nuc, we constructed one Y141A mutant. As observed for many other HUH-endonucleases [39], [40], our *in vitro* cleavage assay results showed that mutation of Tyr141 completely abolished the catalytic activity of NS1_Nuc (Fig. 6A). These observations suggested that NS1_Nuc follows one conserved mechanism in DNA cleavage (Fig. 6B). The DNA cleavage activity of NS1 is cation-dependent. Cation is not present in our structures, but it has been observed in the reported 6USM and 7SZX structures. The side chain of Glu72, His81 and His83 coordinate with cation, which will in turn coordinate and fix the conformation of the phosphate group of C + 1. The side chain hydroxyl group of Tyr141 is very close to C + 1 phosphate group. Once activated, it will attack the phosphorus atom and break the O—P bond between A-1 and C + 1.

Although NS1_Nuc shares conserved mechanism in cleavage, structural comparison suggested that the DNA binding mode of NS1_Nuc is likely different from other HUH-endonuclease family proteins (Fig. 5). To further confirm this hypothesis, we designed several DNA variants (Supplementary Fig. S3A). The DNA-1 sequence is directly derived from the top strand of the DNA Ori of B19V. In the DNA-3 and DNA-4 variants, 3 and 6 nucleotides are simultaneously mutated, at the −9 to −7 and −9 to −4 regions, respectively. Besides the −9 to −4 region, nucleotides at the + 3 to + 5 region are also substituted in the DNA-5 variant. Using WT NS1_Nuc and the DNA variants, we performed *in vitro* cleavage assays. As depicted in Supplementary Fig. S3B, NS1_Nuc has similar cleavage activities on DNA-1, DNA-3, DNA-4 and DNA-5, indicating that the identities of the nucleotides at the −9 to −4 and + 3 to + 5 regions are not critical for ssDNA cleavage by NS1_Nuc. Different from A + 1 and T-4 in the target DNA of WDV Rep (Fig. 5C), our *in vitro* cleavage assay results also suggested that C + 1 and G-4 of NS1_Nuc target DNA do not form stable Watson-Crick pairing.

In addition to DNA, we also purified several mutants of NS1-Nuc (Supplementary Fig. S4) and performed *in vitro* binding and cleavage assays. As depicted in Supplementary Fig. S5, Ala substitution of Leu121 has no obvious impact on DNA binding and cleavage by NS1-Nuc. In contrast to Leu121, single or double mutation of Lys117 and Lys119 lowered the DNA binding and cleavage activities of NS1-Nuc (Fig. 6C-D). Based on structural analyses, protein and DNA mutagenesis and *in vitro* cleavage assay results, we proposed one plausible ssDNA binding model for NS1_Nuc (Fig. 6E). Instead of ‘*n*-shaped’ conformation, the ssDNA adopts a ‘λ-shaped’ conformation. Via their backbone phosphate groups, the nucleotides at the −6 to −4 position may form electrostatic interactions with the side chains of Lys117 and Lys119, which are conserved in NS1.

## 4 Conclusions

B19V NS1 is a multifunctional protein. Via binding and operating on the p6 promoter, NS1 controls the transcription of B19V genome. The null mutants of B19V NS1 completely abolish the infectivity of the virus [20]. NS1 also plays critical roles in viral DNA replication, viral DNA packaging, DNA damage response [42], viral and cellular gene transactivation, cell cycle arrest [21] and modulation of host innate immunity [43]. NS1 is a multidomain protein (Fig. 1A). The TAD subdomain has been confirmed important for the transactivation activity of NS1. In this study, we determined two high-resolution crystal structures of NS1-Nuc. Compared to previous structures, our structures provide more insights into conformational changes, ssDNA binding and cleavage mechanism of NS1. Cleavage at the TRS site of DNA Ori is critical for the replication of B19V genome. Though NS1 shares conserved mechanism in ssDNA cleavage with other HUH-endonucleases, our structural analyses and cleavage assay results support one unique ssDNA binding model for NS1_Nuc (Fig. 6E). Lys117 and Lys119 are involved in DNA binding. The functional importance of Lys117 and Lys119 can be supported by our mutagenesis and *in vitro* DNA binding and cleavage assay results (Fig. 6C-D). The structural basis for A-1, C + 1 and C + 2 selection is not clear at present.

Although B19V is a single-stranded DNA virus, both 5′- and 3′-ends of B19V genome form hairpin-like structures, due to the presence of ITRs. NS1_Nuc can only bind and cleave DNA in single-stranded form. Formation of hairpin structures prevents NS1_Nuc from binding and cleavage at the TRS site. Our *in vitro* binding assay showed that presence of NSBE duplex enhances DNA Ori binding by NS1_Nuc (Fig. 2B-C), and structural comparison revealed several residues critical for NSBE duplex binding by NS1_Nuc (Fig. 4E-H). NS1_Nuc is distantly related to AAV5 Rep and many other Rep proteins, which bind DNA duplex in a cooperative manner. DNA Ori of B19V contains four NSBE elements; theoretically, it can bind four or more NS1_Nuc molecules. Beside nuclease domain, each NS1 molecule also contains one predicted helicase domain, which is likely responsible for unwinding of B19V DNA Ori. In the future, it is worthy to further investigate the structure and DNA unwinding mechanism of NS1 helicase domain.

## Funding

This work was supported by the National Natural Science Foundation of China (32171197 and 31870721).

## CRediT authorship contribution statement

**Yixi Zhang:** Data curation, Visualization, Writing – original draft. **Zhiwei Shao:** Data curation, Methodology. **Yanqing Gao:** Software, Methodology. **Boming Fan:** Data curation, Methodology. **Jie Yang:** Data curation, Software. **Xi Chen:** Data curation, Methodology. **Xin Zhao:** Data curation, Methodology. **Qiyuan Shao:** Data curation, Methodology. **Weizhen Zhang:** Data curation, Methodology. **Chulei Cao:** Data curation, Methodology. **Hehua Liu:** Funding acquisition, Writing – original draft. **Jianhua Gan:** Conceptualization, Funding acquisition, Supervision, Writing – original draft, Writing – review & editing.

## Declaration of Competing Interest

The authors declare that they have no known competing financial interests or personal relationships that could have appeared to influence the work reported in this paper.
