# Supplementary material for: Structures and implications of the nuclease domain of human parvovirus B19 NS1 protein
Source: Comput Struct Biotechnol J. 2022 Aug 27;20:4645–55. doi: 10.1016/j.csbj.2022.08.047 (PMC9440244; doi:10.1016/j.csbj.2022.08.047)
Supplement: Supplementary data 1 [file mmc1.pdf]

Supporting Information

For

Structures and implications of the nuclease domain of human parvovirus B19 NS1 protein

Yixi Zhang[1], Zhiwei Shao[1], Yanqing Gao[1], Boming Fan[1], Jie Yang[1], Xi Chen[1], Xin Zhao[1], Qiyuan Shao[1], Weizhen Zhang[1], Chulei Cao[1], Hehua Liu[1], Jianhua Gan[1,*]

[1]Shanghai Public Health Clinical Center, State Key Laboratory of Genetic Engineering, Collaborative Innovation Center of Genetics and Development, Department of Biochemistry and Biophysics, School of Life Sciences, Fudan University, Shanghai 200438, China.

***Correspondence to**: ganjhh@fudan.edu.cn

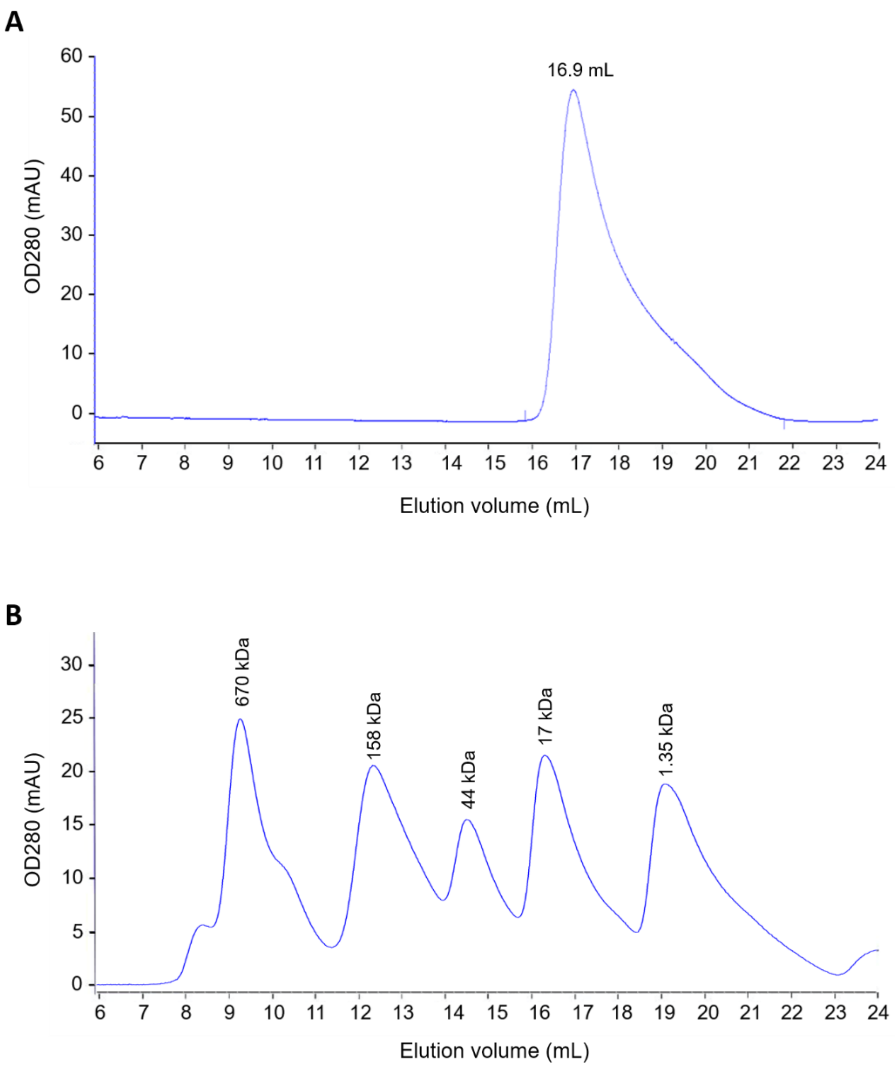

Supplementary Fig. S1. Size-exclusion analysis of (**A**) the NS1_Nuc protein and (**B**) the standard marker protein on Superdex 200 Increase 10/300 GL column.

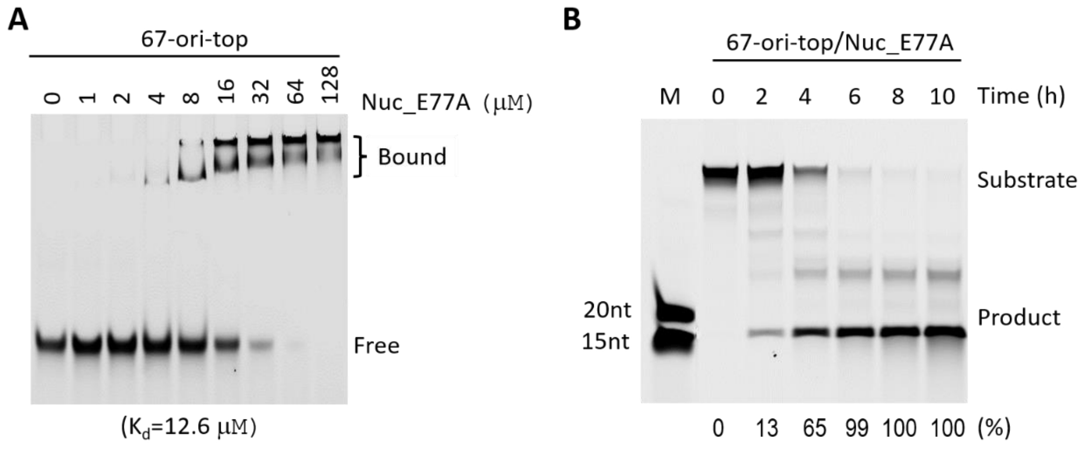

Supplementary Fig. S2. *In vitro* DNA binding (**A**) and cleavage (**B**) by E77A mutant of NS1_Nuc. The K_d value and substrate cleavage percentage derived from three independent experiments are listed at the bottom of the figures.

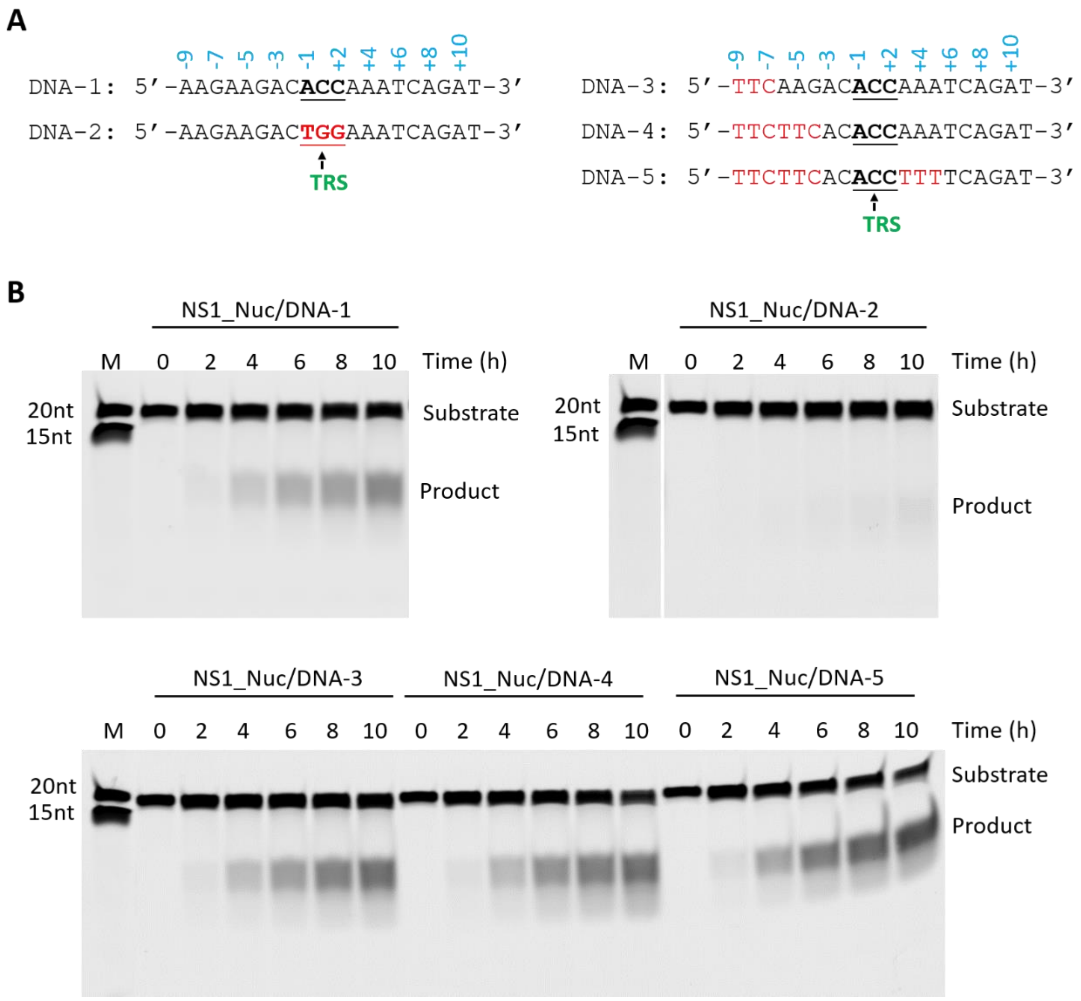

Supplementary Fig. S3. Impacts of DNA mutation on cleavage by NS1-Nuc. (**A**) Detailed sequence of the DNA variants. (**B**) *In vitro* DNA cleavage assays catalyzed by WT NS1_Nuc.

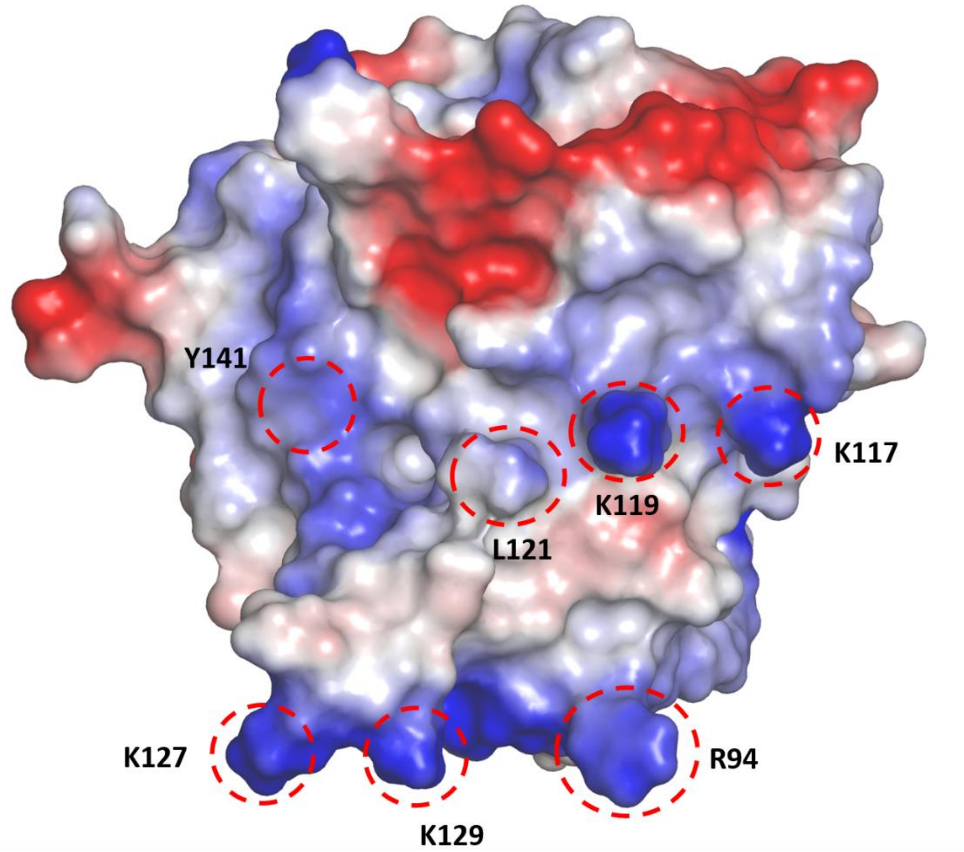

Supplementary Fig. S4. Surface presentation of NS1-Nuc. The residues mutated in this study are highlighted by red circles. The electrostatic surface is calculated by Pymol with the default settings.

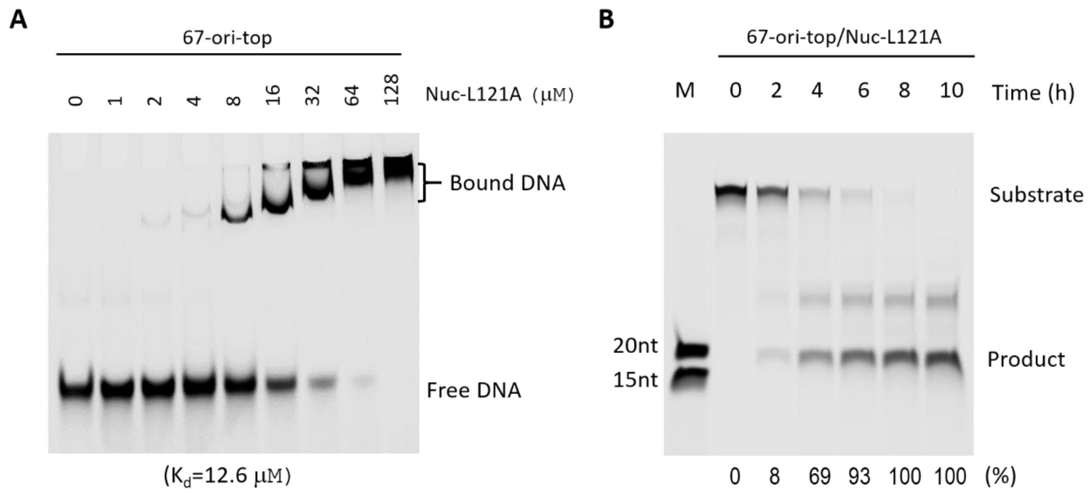

Supplementary Fig. S5. *In vitro* DNA binding (A) and cleavage (B) by L121A mutant of NS1_Nuc. The K_d value and substrate cleavage percentage derived from three independent experiments are listed at the bottom of the figures.

Supplementary Table S1. Codon optimized cDNA sequence of NS1_Nuc (aa 2-176)

The optimized cDNA sequence of NS1_Nuc (from 5' to 3') [a,b]
GGATCC***GGCGGCGGT***GAACTGTTTCGCGGCGTGTTACAGGTGAGTAGCAATGTGCT<br>GGATTGTGCCAATGATAATTGGTGGTGTAGCCTGCTCGACCTGGATACCAGCGATT<br>GGGAACCGTTAACCCATACAAATCGCCTCATGGCCATTTATCTGTCAAGCGTTGCA<br>AGTAAACTGGATTTTACCGGCGGTCCGTTAGCAGGTTGTCTGTATTTTTTTCAGGT<br>GGAATGTAATAAATTTGAAGAAGGCTATCATATTCATGTGGTGATTGGCGGTCCGG<br>GTCTGAATCCGCGCAATCTGACCGTTTGTGTGGAAGGCTTATTTAATAATGTGCTG<br>TATCATTTAGTGACCGGCAATGTTAAACTGAAATTTCTGCCGGGTATGACCACCAA<br>AGGTAAATATTTTCGCGATGGCGAACAGTTTATTGAAAATTATCTGATGAAAAAAA<br>TTCCGCTGAATGTTGTTTGGTGTGTGACCAATATTGATGGCTATATTGATACCTGT<br>ATTAGCGCAACCTTTCGTCGCGGCGCCTGTCATGCCTAA***CTCGAG***

[a]: ***GGATCC*** and ***CTCGAG*** at the 5'-end and 3'-end are Bam HI and Xho I recognition sequence.

[b] ***GGCGGCGGT***, which codes for three Gly residues, was designed to enhance the cleavage efficiency of the UIP1 protease.

Supplementary Table S2. Primers used for NS1_Nuc mutant construction.

Name	Sequence (from 5' to 3')
NS1-Nuc_F	GGATCCGGCGGCGGTGAACTGTTTCGCGGCGTGTTACAGGTG
NS1-Nuc_R	CTCGAGTTAGGCATGACAGGCGCCGCGACGA
E77A_F	GTGGAATGTAATAAATTTGCAGAAGGCTATCATATT
E77A_R	ATGAATATGATAGCCTTCTGCAAATTTATTACATTC
R94A_F	CCGGGTCTGAATCCGGCAAATCTGACCGTTTGT
R94A_R	ACAAACGGTCAGATTTGCCGGATTCAGACCCGG
K117A_F	TTAGTGACCGGCAATGTTGCCCTGAAATTTCTGCCG
K117A_R	ACCCGGCAGAAATTTCAGGGCAACATTGCCGGTCAC
K119A_F	ACCGGCAATGTTAAACTGGCCTTTCTGCCGGGTATG
K119A_R	GGTCATACCCGGCAGAAAGGCCAGTTTAACATTGCC
K117/119A_F	GTGACCGGCAATGTTGCCCTGGCCTTTCTGCCGGGTATG
K117/119A_R	CATACCCGGCAGAAAGGCCAGGGCAACATTGCCGGTCAC
L121A_F	AATGTTAAACTGAAATTTGCACCGGGTATGACCACC
L121A_R	TTTGGTGGTCATACCCGGTGCAAATTTCAGTTTAAC
K127/129A_F	CCGGGTATGACCACCGCCGGTGCCTATTTTCGCGAT
K127/129A_R	GCCATCGCGAAAATAGGCACCGGCGGTGGTCATACC
